# Case Report: Primary hepatic neuroendocrine tumor: two cases report with literature review

**DOI:** 10.3389/fonc.2023.1225583

**Published:** 2023-08-04

**Authors:** Yongsheng Tang, Xianyu Chen, Xu Lu, Zenan Yuan, Yang Yang, Chunhui Qiu, Hua Li

**Affiliations:** ^1^ Department of Hepatic Surgery, Liver Transplantation Center, The Third Affiliated Hospital of Sun Yat-sen University, Guangzhou, China; ^2^ Department of Hepatobiliary Surgery, The Third Affiliated Hospital of Sun Yat-sen University, Guangzhou, China

**Keywords:** TAE, immunohistochemistry, primary hepatic neuroendocrine tumors (PHNETs), PET-CT, liver metastasis, neuroendocrine tumor

## Abstract

**Background & Aims:**

Primary hepatic neuroendocrine tumors (PHNETs) are rare malignant liver tumors that present diagnostic challenges owing to their rarity and absence of specific clinical features. This study aimed to investigate the characteristics of this rare liver tumor to enhance our understanding of the disease, improve diagnostic accuracy, and explore standardized diagnostic and treatment approaches.

**Case description:**

During physical examination, two elderly women, aged 64 and 74 years, were found to have liver masses. 18F-FDG Positron Emission Tomography-Computed Tomography (18F-FDG PET-CT) and Ga68-DOTATATE PET-CT scans of both individuals revealed multiple liver masses that were initially suspected to be hepatic neuroendocrine tumors. Subsequent puncture pathology confirmed the diagnosis of neuroendocrine tumors. Furthermore, in Case 1, the tumor was also detected by 18F-FDG PET-CT in the lung, suggesting a metastatic tumor, in conjunction with liver immunohistochemistry and imaging findings. Laboratory tests revealed no significant abnormalities in liver function or autoimmune liver disease indicators, and there was no evidence of viral hepatitis infection. However, partial hepatectomy was not indicated for cases with distant metastasis or multiple space-occupying lesions. Individualized treatment approaches have been developed for such situations. A large portion of the tumor underwent Transarterial Embolization (TAE), and targeted combination chemotherapy or endocrine therapy was administered based on the pathological results. During regular follow-ups a 13 and 12 months, the tumor remained stable. The patients’ quality of life was good, and their psychological well-being was healthy. They led active lifestyles, demonstrated a thorough understanding of their disease and its progression, and actively cooperated during the follow-up process.

**Conclusion:**

Our findings suggest that a combination of serological, radiological, and immunohistochemical examinations can aid in the diagnosis of PHNET. In addition, we determined that TAE combined with drug therapy could be an effective method for controlling PHNET progression. Regular postoperative follow-ups are important for monitoring the prognosis and tumor progression status of patients with PHNET.

## 1 Introduction

Neuroendocrine tumors (NETs) are rare malignancies originating from the neuroendocrine system cells (1). They are most commonly found in organs such as the gastrointestinal and respiratory tracts (2). Primary hepatic neuroendocrine tumors (PHNETs) are uncommon and have a slow growth rate, which often makes them difficult to detect until the disease progresses to a later stage (3). Due to their nonspecific radiographic features, diagnosing PHNETs can be challenging and often leads to confusion with other types of liver lesions. However, histopathology and immunohistochemistry can aid in the accurate diagnosis of PHNETs. This study aimed to examine the clinical presentation, pathological characteristics, therapeutic approaches, and prognoses of two patients who were diagnosed with PHNETs and received treatment at our medical center.

## 2 Case presentation

### 2.1 Case 1

#### 2.1.1 History and clinicoradiological evaluations

A 64-year-old female patient with a medical history of hypertension, diabetes, and hyperlipidemia was admitted to our hospital, and a liver mass was discovered during a routine health check-up a month prior. Routine tests, including alpha-fetoprotein (AFP), carcinoembryonic antigen (CEA), and cancer antigen 199 (CA199) tests, showed normal results, and the basic diseases were well controlled. Hepatitis B surface antigen and hepatitis C antibody test results were negative.

**Figure 1:**
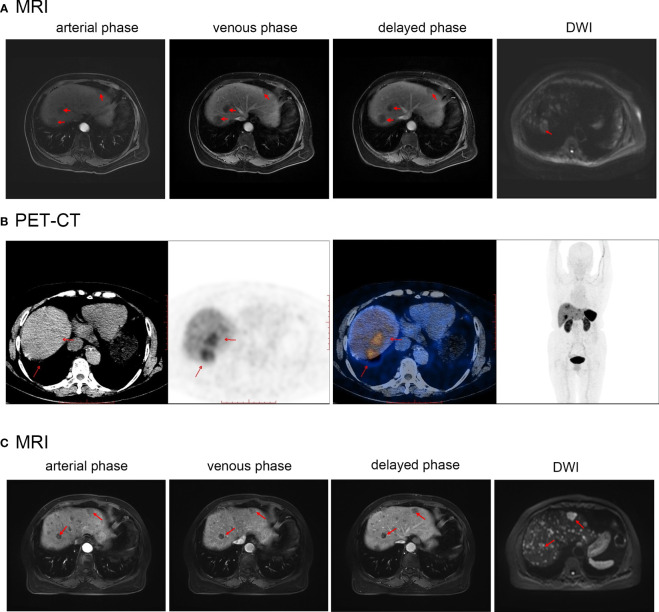
**(A)** Abdominal and chest CT before first treatment of Case1. **(B)** PET-CT before the first treatment of Case1. **(C)** Abdominal and chest CT after the first treatment of Case1. Red arrows indicate the location of the tumor.

Abdominal computed tomography (CT) (**Figure 1A**) indicated the presence of a mass of approximately 40 × 35 mm in size with an irregular shape in the S5/8 segment of the liver. The mass showed peripheral ring enhancement during the arterial phase, but decreased enhancement during the portal and delayed phases. Invasion was suspected in the portal branch of segment 8 and hepatic central vein. A chest CT scan (**Figure 1A**) demonstrated an irregular soft tissue mass with a circular shape in the lower lobe of the right lung, indicating a possible metastatic lesion. 18F-FDG PET-CT (**Figure 1B**) demonstrated the presence of several hypodense lesions with ill-defined margins and elevated glucose uptake in the liver, and a nonuniform solid mass with increased glucose metabolism in the lungs.

#### 2.1.2 Treatment and postoperative course

Radical resection could not be performed because of the presence of distant metastases and multiple intrahepatic lesions. The patient underwent S5/8 TAE, followed by chemotherapy with a combination of capecitabine and temozolomide. TAE is a routine procedure performed at our center. Before the operation, the cardiopulmonary function test results improved, and there were no obvious contraindications. The patient was treated with TAE consisting of one blank microsphere (40-120 µm) and one bottle of gelatin sponge (150 µm) were mixed with contrast agent as an embolic agent. After the operation, mild liver function abnormalities soon returned to normal levels. A follow-up CT scan showed that most of the S5/8 liver lesion had been necrotized, and the size of the lung mass was essentially unchanged (**Figure 1C**). Nine months after receiving the regimen, a CT scan performed during follow-up showed that some liver lesions had increased in size, whereas the right lower lobe lung mass remained similar to previous measurements. The chemotherapy drug was then changed to sunitinib; however, owing to symptoms of dizziness, fatigue, poor appetite, and limb weakness after one month, it was replaced with anlotinib hydrochloride. Follow-up CT revealed a slight decrease in the size of the liver lesions and lung mass. The patient was followed-up for 13 months. The disease was stable with no significant progression, the patient ‘s quality of life was good, they fully understood their own disease, and they cooperated with the treatment and follow-up.

#### 2.1.3 Histopathological examination

**Figure 2:**
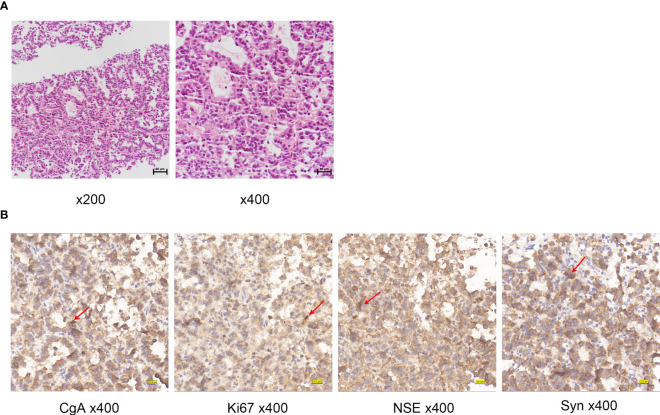
**(A)** Histologic examination of tumor tissues by hematoxylin and eosin staining of Case1. **(B)** Immunohistochemistry of Case1: CgA, NSE, Ki67, Syn. Red arrows indicate positive cells.

A liver biopsy was performed and pathological analysis (**Figure 2A**) revealed tumor cells arranged in a nest-like pattern, exhibiting mild-to-moderate atypia, an abundant cytoplasm containing fine granules, and delicate chromatin. Immunohistochemical analysis (**Figure 2B**) revealed that the tumor cells were positive for synaptophysin (Syn), chromogranin A (CgA), and neuron-specific enolase (NSE). The Ki67 proliferation index was 25%, indicating high nuclear reactivity and a greater likelihood of aggressive tumor behavior. A Grade 3 liver neuroendocrine tumor was diagnosed.

### 2.2 Case 2

#### 2.2.1 History and clinicoradiological evaluations

During a routine medical examination, a 74-year-old female patient was found to have a liver mass and was admitted for treatment. The patient had a medical history of diabetes and hypertension, but did not exhibit any symptoms, such as abdominal pain, bloating, diarrhea, nausea, vomiting, skin jaundice, or itching. Various parameters, such as AFP, CA199, CEA, and cancer antigen 125 (CA125), were within normal limits, and basic diseases were well controlled. Serological testing also did not detect any evidence of hepatitis B or C virus infection, and liver function was within the normal range.

**Figure 3:**
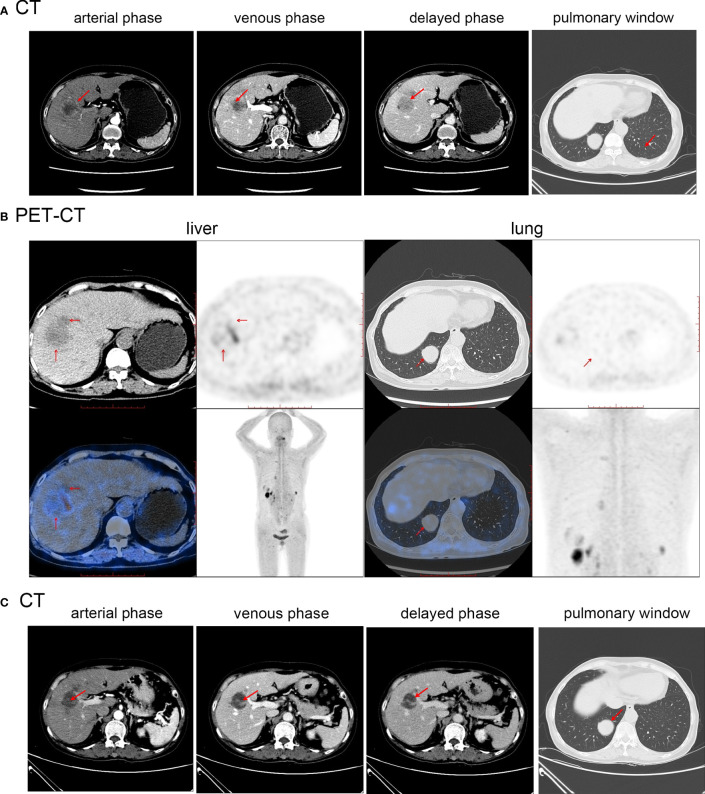
**(A)** Abdominal MR before first treatment of Case2. **(B)** Abdominal PET-CT before initial treatment of Case2. **(C)** Abdominal MR after first treatment of Case2. Red arrows indicate the location of the tumor.

Abdominal MRI revealed several liver masses (**Figure 3A**). The largest mass, measuring 25 × 24 mm in the S7/8 segment, exhibited slightly longer T1 and T2 signals and high signal intensity on diffusion-weighted imaging (DWI). No filling defects were observed in the main portal veins or branches. 18F-FDG PET-CT performed in other hospitals before admission did not have a good differential effect; therefore, Ga68-DOTATATE PET-CT was performed, which showed a circular area with increased radioactive uptake (maximum standardized uptake value:24.5), consistent with the appearance of a neuroendocrine tumor in this region (**Figure 3B**). No neuroendocrine tumors were detected in other parts of the body during imaging studies.

#### 2.2.2 Treatment and postoperative course

Radical resection could not be performed due to the presence of multiple intrahepatic lesions. Following confirmation of the diagnosis, a S7/8 TAE procedure was performed, and the patient was treated with TAE consisting of blank embolization microspheres (100-300 µm) were mixed with contrast agent. The patient received subcutaneous injections of 0.1mg octreotide acetate three times a day postoperatively. Following discharge, the patient was administered a monthly injection of a 20 mg octreotide acetate depot. Two months later, a follow-up MRI (**Figure 3C**) showed a decrease in the size of the largest tumor in the S7/8 segment to 17×16 mm, while the tumor size in the S4 segment remained relatively unchanged (17.8×19.9 mm). The patient underwent S4 TAE surgery and was subsequently prescribed a monthly intramuscular injection of octreotide acetate microspheres (20 mg). At present, patient has been followed-up for 12 months, and no progression of the tumor or development of new lesions has been observed. The patient remains asymptomatic. The patient has a good mentality, lives an active lifestyle, understands the condition and development, and actively cooperates with follow-up treatment.

#### 2.2.3 Histopathological examination

**Figure 4:**
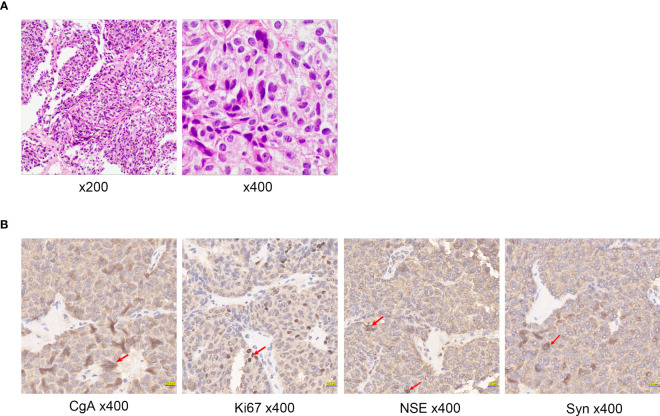
**(A)** Histologic examination of tumor tissues by hematoxylin and eosin staining of Case2. **(B)** Immunohistochemistry of Case2: CgA, NSE, Ki67, Syn. Red arrows indicate positive cells.

The pathology report from the liver biopsy as well as immunohistochemistry (**Figure 4A**) staining revealed widespread expression of CgA, Syn, and NSE in the tumor cells (**Figure 4B**). The tumor was classified as a grade 2 neuroendocrine tumor based on the presence of up to three mitotic figures per 10 high-power fields and a Ki-67 index of 15%.

## 3 Literature review

We conducted a literature review to identify the cases reported over the past 15 years. A systematic search using the term ‘Primary Hepatic Neuroendocrine Tumor’ was performed in the PubMed database. This search yielded 31 publications reporting 43 cases. Among the patients, 15 (35%) were male and 28 (65%) were female. The tumors were located in the right lobe of the liver in 22 cases (51%), the left lobe in 12 cases (28%), and both lobes in nine cases (21%). Abdominal pain was the primary complaint in 19 patients (44%), whereas 11 (26%) cases were incidentally detected during routine check-ups. Additionally, two cases presented mainly with abdominal distension, one case with an abdominal mass, and one with predominant symptoms of nausea and vomiting. Of the total cases, 31 (72%) underwent liver resection; and among them, 21 showed no recurrence during the follow-up period. Moreover, TACE yielded promising outcomes in Case 17, 19, 26, and 37, demonstrating its feasibility as a therapeutic approach. In Case 27 and 36, chemotherapy drugs and somatostatin analogs showed potential therapeutic value for hepatic neuroendocrine tumors. Case 41 underwent TAE alone, and no tumor recurrence was observed during the 6-month follow-up period. Notably, Case 36, who received a combination of surgical intervention and somatostatin analogs, exhibited a remarkable survival time exceeding 16 months. Case 23 underwent a combination of liver resection and transplantation, resulting in an impressive survival time of > 14 years. Similarly, Case 7 underwent liver resection combined with multiple TACE procedures and achieved a survival time exceeding 25 months. Additionally, Case 8 and 28 were treated with a combination of liver resection and TACE, leading to postoperative survival times exceeding 27 and 12 months, respectively. These findings suggest that combination therapy has great potential as a beneficial treatment approach for hepatic neuroendocrine tumors. The demographic and clinical profiles are summarized in **Table 1**.

**Table 1:** The summary of demographic and clinical data.

Author/year	NO.	Age/sex	Max size (cm)	Location	Symptoms	Treatment	Survival months	Tumor grade
Skagias, L. et al. (4)/2010	1	74/M	15	Left	Abdominal pain	Hepatectomy	N/D	N/D
Jia, C. et al. (5)/2012	2	N/D	3.7	Left	N/D	Hepatectomy	>24	N/D
	3	N/D	14.1	Left and right	N/D	Chemotherapy	7	N/D
	4	N/D	4.1	Right	N/D	Hepatectomy	>51	N/D
	5	N/D	3	Left	N/D	Hepatectomy	>37	N/D
	6	N/D	4.1	Right	N/D	Radiotherapy	N/D	N/D
	7	N/D	2.7	Right	N/D	TACE, hepatectomy, TACE	>25	N/D
	8	N/D	3.4	Right	N/D	Hepatectomy, TACE	>27	N/D
	9	N/D	1.8	Left and right	N/D	Radiofrequency ablation	1	N/D
	10	N/D	13	Right	N/D	Hepatectomy	N/D	N/D
Yalav, O. et al. (6)/2012	11	43/F	10	Left	Abdominal pain	Hepatectomy	N/D	N/D
	12	34/M	18	Right	Abdominal pain	Hepatectomy	N/D	N/D
	13	50/F	10	Left and right	Abdominal pain	Hepatectomy	N/D	N/D
	14	41/M	15	Right	Abdominal pain, fever	Hepatectomy	N/D	N/D
	15	42/M	20	Left	Abdominal pain	Hepatectomy	N/D	N/D
Baek, S. H. et al. (7)/2013	16	71/M	3.7	Left	incidental	Hepatectomy	>36	G2
Yang, K. et al. (8)/2015	17	41N/D	2.8	Right	Abdominal pain	TACE	>14	G2
Mitamura, K. et al. (9)/2015	18	70/M	23	Right	hepatic mass	Hepatectomy	>24	G1
Morishita, A. et al. (10)/2016	19	87/M	2	Right	incidental	TACE	21	N/D
Song, J. et al. (11)/2016	20	70/M	8.3	Right	Abdominal pain	Hepatectomy	>24	G2
Hu, X. et al. (12)/2016	21	41/F	11.2	Right	incidental	Hepatectomy	N/D	G2
Sethi, S. et al. (2)/2016	22	35/N/D	3.7	Left	cough	Hepatectomy	>36	N/D
Ibrahim, M. et al. (13)/2017	23	57/F	14.5	Left and right	Incidental	Hepatectomy, liver transplantation, hepatectomy	>144	G1 to 2
Gorla, A. et al. (14)/2017	24	17/M	N/D	Right	Abdominal pain	Hepatectomy	>34	N/D
Meng, X. et al. (15)/2018	25	56/F	26	Right, involvement of blood vessels	Abdominal distension, abdominal pain	Hepatectomy	>72	G1
Bai, X. et al. (16)/2019	26	53/M	N/D	Right	Abdominal bloating, nausea, poor appetite, fever	TACE	>6	N/D
Jain, R. et al. (17)/2020	27	38/F	N/D	Left, involvement wall of the gallbladder	Abdominal pain, loss of weight	Chemotherapy	>3	G3
Xia, Y. et al. (18)/2020	28	38/M	10	Left, right, caudate, Involvement of blood vessels	Abdominal pain	Hepatectomy, TACE	>12	G2
Li, Z. et al. (19)/2020	29	72/F	1.5	Right	Incidental	Hepatectomy	>48	G3
Costa, A. et al. (20)/2020	30	51/F	5	Left	Abdominal pain	Hepatectomy	>12	G1
Liu, F. et al. (21)/2020	31	78/F	6.2	Left	incidental	Hepatectomy	N/D	G2
Huang, H. et al. (22)/2020	32	42/M	14	Right, involvement of blood vessels	Abdominal pain	Hepatectomy, chemotherapy	N/D	G3
Alakeel, A. et al. (23)/2020	33	41/F	15.1	Left and right	Abdominal pain	TACE, hepatectomy	>2	G2
Tuan Linh. et al. (3)/2021	34	57/M	4	Left and right	Abdominal pain, diarrhea	N/D	>15	G2
Kim, J. et al. (24)/2021	35	51/F	14.7	Left	Abdominal discomfort, dyspnea	Hepatectomy	N/D	G2
Wong, P. et al. (25)/2021	36	69/M	7.1	Left, right Involvement of blood vessels and bile duct	Incidental	Hepatectomy, lanreotide	>16	G2
Rao, Y. et al. (26)/2021	37	79/M	4	Right	incidental	TACE	>18	G2
Chatziioannou. et al. (27)/2022	38	45/F	36	Left and right	incidental	Liver transplantation	>5	G2
Waghela, R. et al. (28)/2022	39	63/F	4.8	Right	Nausea, vomiting	Hepatectomy, TARE, chemotherapy	2	G2
Andriani, J. et al. (29)/2022	40	19/F	10	Right	Vomiting, abdominal pain, short breath, generalized weakness	Hepatectomy	>12	G2
Andriani, J. et al. (29)/2022	41	18/F	18	Right	Abdominal pain, anemia	TAE	>6	N/D
Song, S. et al. (30)/2022	42	22/M	5	Right	Abdominal pain	Hepatectomy	Recovered	G1
Zhao, B. et al. (31)/2023	43	26/M	7.1	Left	Incidental	Hepatectomy	>12	G2

M, male; F, female; N/D, no data; TAE, transcatheter arterial embolization; TACE, transarterial chemoembolization; TARE, Transarterial radioembolization.

## 4 Discussion

Primary hepatic neuroendocrine tumors (PHNETs) are rare, as most hepatic neuroendocrine tumors originate from metastases to other organs (2). The exact origin of PHNETs remain uncertain, but they are believed to arise from neuroendocrine cells in the biliary epithelium of the liver, ectopic adrenal or pancreatic tissue, or stem cells in the liver (3). Owing to the limited understanding of the clinical characteristics and treatment modalities off PHNETs, there has been an ongoing debate regarding its diagnosis. Therefore, comprehensive reports on PHNETs are essential to improve our understanding of this condition.

Studies have shown that PHNET primarily affects middle-aged individuals and have a higher incidence in females (3). The most common clinical symptom of PHNETs is caused by the compression of late-stage tumors, including abdominal pain, abdominal masses, and jaundice (32). It is also common for hepatic lesions to be discovered during physical examinations without any symptoms. The lesions were discovered during a routine physical examination of the patients reported in this article. Symptoms of carcinoid syndrome may be present, including facial flushing, shortness of breath, and diarrhea (32, 33). CT examination is a useful tool for detecting PHNETs, which typically appear as low-density masses with significant enhancement in the arterial phase and reduced enhancement in the portal or delayed phases. In a study of 38 cases of PHNETs, researchers analyzed dynamic enhanced CT characteristics and found that 74% of the cases showed enhancement during the arterial phase, 52% showed delayed enhancement, and 48% exhibited a pattern of enhancement similar to that of hepatocellular carcinoma (34). MRI of PHNETs typically shows ring-like enhancement during the arterial phase and persistent enhancement in the portal or delayed phase, with high signal intensity observed on DWI sequence (35). When CT or MRI cannot distinguish PHNETs from other liver malignancies, PET-CT, such as 18F-FDG and Ga68-DOTATATE PET-CT, provide more evidence for diagnosis. Ga68-DOTATATE PET-CT, with its higher sensitivity and spatial resolution, is a promising diagnostic tool for the diagnosis of neuroendocrine tumors, and provided a clear diagnosis in our cases.

Diagnosing PHNETs can be challenging, as relying solely on imaging may not be sufficient to distinguish them from other malignant liver tumors. Therefore, thorough medical history and serum tests are necessary for an accurate diagnosis. For example, primary liver cancer often presents with a history of hepatitis B-related cirrhosis, and an elevated AFP level is a crucial diagnostic indicator that is not particularly useful in the case of PHNETs. In both cases, we did not observe any abnormalities in tumor markers, hepatitis B and C antigens, or liver function tests. However, to diagnose primary hepatic neuroendocrine tumors, it is crucial to rule out the presence of neuroendocrine tumors in other organs. When both the liver and lungs are affected, the origin of the tumor can be confirmed using immunohistochemical markers. These markers, including Syn, CgA, CD56, and NSE, are highly sensitive tools for diagnosing NETs in any part of the body and are widely accepted in the medical community (3, 36). A positive CDX2 result indicates that the tumor may have originated from the gastrointestinal tract, which can help exclude the possibility of a primary lung lesion. Conversely, TTF-1 positivity may suggest a thoracic tumor origin (37). NETs can be classified based on their mitotic rate and Ki-67 proliferation index (38). The WHO grading system divides PHNET into three grades: G1, which has a mitotic count of less than 2/10 HPF and/or a Ki-67 index of ≤2%; G2, which has a mitotic count of 2-20/10 HPF and/or a Ki-67 index of 3-20%; and neuroendocrine carcinoma (NEC), which has a mitotic count of > 20/10 HPF and/or a Ki-67 index of > 20%.

Currently, owing to the limited number of cases, there are no established treatment guidelines for PHNETs. However, surgical resection (specifically anatomical liver resection) was considered the treatment of choice and can potentially lead to a complete cure, with a reported 5-year survival rate of 78% (39, 40). In cases where primary lesions are deemed unresectable, alternative options, such as arterial embolization, chemotherapy, radiotherapy, somatostatin analogs, conservative therapy, and liver transplantation, can be considered after exhausting all other measures (41–45). Among the reported cases, two patients with PHNETs underwent TAE; in Case 1, TAE was combined with chemotherapy and targeted therapy, whereas in Case 2, it was combined with octreotide treatment after surgery. Currently, both patients have maintained a good quality of life without significant disease progression. These findings suggest that when liver resection is not feasible, the combination of TAE and drug therapy has the potential to control the progression of PHNET, although it may not be as effective as liver transplantation.

## 5 Conclusion

PHNETs are rare liver tumors that often present with nonspecific symptoms, making it challenging to distinguish them from other types of liver tumors. Ga68-DOTATATE PET-CT imaging can provide valuable evidence for the diagnosis of PHNETs; however, it should be complemented by a comprehensive evaluation of serology and immunohistochemistry. Hepatectomy is the preferred treatment modality for PHNETs. However, in cases where multiple intrahepatic lesions are present or vascular invasion cannot be effectively treated with surgery, alternative approaches must be considered. Although the efficacy of TAE combined with chemotherapy and targeted therapy or TAE combined with endocrine therapy may not be as significant as that of liver transplantation, these interventions still have the potential to control the progression of PHNETs.

## Data availability statement

The original contributions presented in the study are included in the article/**Supplementary Material**. Further inquiries can be directed to the corresponding authors.

## Ethics statement

This study was approved by the Medical Ethics Committee of the Third Affiliated Hospital of Sun Yat-sen University, under the registration number:CR2023-001-01. Written informed consent was obtained from the patient for publication of this case report and accompanying images. A copy of the written consent is available for review by the Editor-in-Chief of this journal on request.

## Author contributions

Study concept and design: HL, CQ, and YT. Data collection: YT, XC, and CQ. Data analysis and interpretation: YT, XC, XL, and ZY. Writing the paper: YT and XC. Revision: XL, ZY, CQ, HL, and YY. All authors contributed to the article and approved the submitted version.
