# Supplementary material for: Case Report: Primary hepatic neuroendocrine tumor: two cases report with literature review
Source: Front Oncol. 2023 Aug 4;13:1225583. doi: 10.3389/fonc.2023.1225583 (PMC10436565; doi:10.3389/fonc.2023.1225583)
Supplement: Supplementary file 1 [file DataSheet_1.pdf]

Case1

Date	Therapeutic process
2022-4-25	Health examination CT: multiple liver lesions.
2022-4-27	18F-FDG PET-CT: liver S5/8 mass, imaging positive, considering the possibility of primary malignant tumor of the liver.
2022-4-28	CT + enhancement : liver S5/8 mass (about 40×35mm) and S4, S4/8, S8 nodules The size of the chest CT was about 47×39mm.
2022-4-29	Liver biopsy: TTF-1(-), CgA(+) ,NSE(+) ,Syn(+) , Ki-67 (hot spot about 25%+) neuroendocrine tumors (NET, consider G3).
2022-5-9	TAE: S5/8 tumor was embolized.
2022-5-14	Discharge medication:capecitabine (750mg/m2 bid D1-14) combined with temozolomide (200mg/m2 D10-14).
2022-6-13	Most of the liver S5 / 8 tumor necrosis ;The size of the chest CT is about 47×35 mm, which is not much changed compared with the previous size.
2023-1-12	Some lesions in the liver were larger than before (40*39*38mm), and the mass in the lower lobe of the right lung was similar to that before (32*30*38mm), so it was replaced with sovantinib.
2023-3-29	The side effects were obvious after one month: dizziness, fatigue, anorexia, limb weakness; so it was replaced by anlotinib.
2023-3-29	Reexamination showed that the intrahepatic lesion was smaller than before (34*36* 32mm), and the right lower lung mass was smaller than before (30*25*37mm).
2023-5-1	Liver and lung was stable and had no obvious progress.
2023-6-20	Stable condition and had no obvious progress. The patient 's quality of life is good, fully understand their own disease, cooperate with treatment and follow-up.

Case2

Date	Therapeutic process
2022-5	A liver mass was found by physical examination.
2022-6	MR: liver multiple space-occupying lesions, maximum about 19mm*23mm, consider neuroendocrine tumors?
2022-6	18F-FDG PET-CT: multiple low-density nodules in the liver, and no abnormal increase in FDG metabolism (considering hepatic epithelioid hemangioendothelioma?;Neuroendocrine tumor ?).
2022-6	Ga68-DOTATATE PET/CT: neuroendocrine tumor (S7/8)
2022-6	Liver biopsy: TTF-1 (-); NSE (+), Syn (+), CgA(+), Ki67 (hot spot about 15%+, consider neuroendocrine tumors,G2).
2022-6	TAE treatment was performed on S7/8,at the same time to octreotide acetate (octreotide acetate 1ml:0.1mg) TID subcutaneous injection at the time of discharge to octreotide acetate microspheres (octreotide acetate microspheres) 20mg deep intramuscular injection once a month.
2022-8	Reexamination MR: S7/8 reduced from 25*22mm and 18*19mm to 17*16mm and 13.8* 12mm. S4: 17.8*19.9mm
2022-8	TAE embolization again: S4 embolization and octreotide acetate microspheres (octreotide acetate microspheres) 20mg deep intramuscular injection once a month.
2023-5-1	Tumor did not progress and no new cases were found.
2023-6-20	Tumor is stable and had no obvious progress. The patient 's mentality is good, active life, understand the condition and development, active treatment with follow-up.
